# Exploring the necessity of establishing a doctor of nursing practice program from experts’ views: a qualitative study

**DOI:** 10.1186/s12909-021-02758-w

**Published:** 2021-06-07

**Authors:** Mozhgan Rivaz, Paymaneh Shokrollahi, Elahe Setoodegan, Farkhondeh Sharif

**Affiliations:** 1 Community Based Psychiatric Care Research Center, Department of Nursing, School of Nursing and Midwifery, Shiraz University of Medical Sciences, Shiraz, Iran; 2 School of Medical Science, Islamic Azad University-Firoozabad, Firoozabad, Iran; 3 Student Research Community, Shiraz University of Medical Sciences, Clinical Supervisor of Beheshti Hospital, Iranian Social Security Organization, Shiraz, Iran; 4 Community Based Psychiatric Care Research Center, Shiraz University of Medical Sciences, Shiraz, Iran

**Keywords:** Doctor of nursing practice, Nursing doctorate, Advanced practice nursing, Nursing education, Iran, Qualitative study

## Abstract

**Background:**

Complex healthcare systems increasingly demand influential nurse leaders adept at managing changes in unstable environments. The doctor of nursing practice (DNP) prepares the nurses for the most advanced level of clinical practice. The aim of this study was to explore the necessity of establishing a DNP program in Iran from experts’ views.

**Methods:**

The study used a qualitative descriptive approach. The participants consisted of 13 faculty members and Ph.D. candidates selected using purposive sampling. Data were collected through focus group and semi-structured interviews, and analyzed using qualitative content analysis.

**Results:**

The content analysis led to the extraction of two main categories: “providing infrastructures” and “DNP as an opportunity to make positive outcomes.“

**Conclusions:**

It is concluded that it is not necessary to establish a DNP program for Iran’s nursing education system. Supplying infrastructures is a crucial component to establishing a new program in Iran. Although DNP, as an opportunity to drive positive changes, is recommended, in the current situation, using alternative solutions may yield better outcomes than establishing a DNP program.

**Supplementary Information:**

The online version contains supplementary material available at 10.1186/s12909-021-02758-w.

## Background

Healthcare systems are rapidly evolving. Complex healthcare landscapes increasingly demand influential nurse leaders adept at managing changes in unstable environments [1]. A nursing doctoral degree is necessary to promote autonomy, professional development, and professional positions in the leadership, research, and educational levels [2].

Generally, there are two kinds of terminal degrees in the nursing profession: the Doctor of Philosophy (Ph.D.) and the Doctor of Nursing Practice (DNP). The focus of the Ph.D. degree is on research and knowledge generation, aiming at advancing the theoretical foundations of the profession, while the DNP degree is a practice-focused doctorate. The scope of DNP programs is varied, but “specialty competencies” are the core of all advanced practice roles [3].

DNP is a powerful strategy to prepare nurses for the most advanced clinical nursing practice level, i.e. “providing leadership for evidence-based practice” [4]. It focuses on advanced practice roles, including leadership, management, healthcare policies, interdisciplinary collaboration, clinical reasoning skills, and translation of scientific and theoretical knowledge into practice for solving problems and improving health outcomes [5, 6]. DNP nurses also influence the effectiveness of health systems, health insurance, and health economics through contributing to clinical practice, evaluating the cost-effectiveness of patient care strategies, coordinating quality improvement teams, and analyzing cost-effective protocols [3].

In 2004, the American Association of Colleges of Nursing (AACN) members endorsed the DNP degree for advanced practice registered nurses to better align nursing with other health professionals by 2015 [7]. The National Association of Clinical Nurse Specialists has confirmed DNP’s entry into practice by 2030 [8].

The combined knowledge and skills of both DNP and Ph.D. nurses can decrease the gap between theory and practice. Collaboration among nurses with DNP and Ph.D. degrees to advance the knowledge and translate the research into practice provides educational opportunities, facilitates healthcare delivery, and improves desired outcomes [1].

In Iran, nursing education is a university-based program and includes the Bachelor of Science, the Master of Science, and Ph.D. in nursing. A bachelor’s degree is required for registered nurses, and it is the basic nursing program at the academic level. This program lasts for four years and requires students to complete at least 130 credits of coursework and training. Clinical training starts from the second semester simultaneously with theoretical courses and ends at the end of the sixth semester, and the curriculum is completed in a full-time internship period [9, 10].

MSN programs are 2 to 3 years in length and involve 30 to 44 credits, depending on each university’s policies. Usually, in the third semester, four to six modules are assigned to the dissertation project. An MSN curriculum content focuses on theoretical knowledge, research, statistical analysis, and, to a lesser extent, clinical courses. Although MSN programs aim to develop registered nurses’ knowledge and clinical competency, most of these programs focus on theoretical knowledge rather than practical skills. The greatest incentive for registered nurses to participate in MS programs is financial advantages followed by leaving clinical settings for educational environments [10] due to poor practice environments, inadequate resources, heavy workload, low salaries, inappropriate nurse-patient ratio, and limited clinical autonomy [11, 12].

The first Ph.D. program was launched at the Tabriz University of Medical Sciences, Iran, in 1992. It lasted 4–5 years, and Ph.D. candidates completed 45credits, including 20 credits of dissertation. The program included two courses on education and research [10].

In recent years, the nursing profession in Iran has made significant advancements in various nursing practices, such as hospital-based practice, critical care nursing, primary healthcare, community-based care [13], spiritual care [14], education, and research. However, it has also faced several challenges. The evidence shows limited clinical authority and autonomy, limited collaboration in clinical decision-making, a lack of collaboration in organizational and national healthcare policies, leadership, healthcare marketing, and evidence-based practice [9, 10], and a lack of emphasis on holistic care in nursing education [15] are the most frequent challenges. We need to deal with these challenges to increase nurses’ job satisfaction, support career development programs, and ensure patient safety.

In Iran, doctoral programs in nursing are limited to a Ph.D. degree, and large numbers of nurse educators and Ph.D. candidates seek a doctoral degree for attaining tenure and promotion in universities. There are few clinical positions for Ph.D. graduates and those preferring academic and research roles [16]. In comparison, in developed countries (e.g., the United States), doctoral education in nursing is undergoing a paradigm shift. Over the last eight years, programs offering the DNP degree have been emerging rapidly, and universities have continued to respond to the Institute of Medicine’s 2010 voice to double the number of DNP nurses by 2020 [17].

DNP programs initially started in the United States, Canada, and the United Kingdom. Healthcare systems’ complexity in South and South-East Asian Countries has led to changes in these programs [18]. There is a growing trend in improving postgraduate programs for preparing nurses to meet the health system’s current and future expectations in Iran.

In 2013, AACN reported that 241 colleges and universities across the United States offered DNP degrees [6]. Despite an increasing number of DNP programs in the United States, there is little evidence-based information about the impact of DNP on patient outcomes and the healthcare organization. Also, there is no standardized method or valid instrument for evaluating DNP graduates’ contribution to the nursing profession’s quality of care and healthcare outcomes [19]. Besides, the shortage of faculty members and, consequently, nurses is a serious concern in the United States. Kelly (2010) concluded that DNP was not the answer to the faculty shortage, and that faculty salaries should become more competitive with salaries for clinical positions to attract nurses with advanced roles [20].

There is no consensus on whether DNP programs are required in Iran’s nursing education system; an issue that should be explored by specialists. Due to differences in the social, political, economic, and cultural contexts of the healthcare system in Iran, this qualitative study aimed to explore the necessity of establishing a DNP program in Iran from experts’ views.

## Methods

### Study design

The study used qualitative content analysis with a naturalistic paradigm. The paradigm includes beliefs and values forming a conceptual and theoretical framework to answer research questions [21]. Naturalistic approaches create a deep insight into the experiences of the participants. The qualitative content analysis approach is particularly appropriate for areas in which we have little theoretical or practical knowledge [22]. Since no studies have investigated the necessity of establishing DNP programs, we used qualitative methodology to reach the study’s objective.

### Participants and recruitment

The participants consisted of 13 faculty members and Ph.D. candidates selected using purposive sampling [22]. We selected critical informants with rich information on the research topic based on the individual judgment, the topic, and the following question: Who can give us sufficient, relevant, and deep information about the topic? [23].The inclusion criteria were appropriate knowledge of the topic, willingness to participate, willingness to share experiences, Ph.D. holders, and Ph.D. candidates.

### Data collection

At first, we collected data using focus group discussion with 11 of the participants. Then, we conducted individual semi-structured face-to-face interviews with two professors in nursing education, who were members of the Iranian Board of Nursing from March 2016 to June 2017. The synergistic nature of focus group discussion and its social interactions motivate participants to speak up, producing rich data [24]. The integration of a focus group and individual interviews enhances the data’s richness and improves its trustworthiness [25, 26]. The interviewers followed a guide developed by the research team (Additional File 1). The interviews began with general questions, such as “What do you think about setting up a DNP program in the Iranian nursing education system?” and continued using probing questions, such as “Please explain more,“ “why?“ and “how?“ All interviews were conducted in a peaceful environment in workplace. The interview duration in the focus group and for each individual was 120 min and 30–60 min, respectively. Author 1 and Author 3 conducted all interviews, audio-recorded them, and transcribed the data verbatim. The interviewers were PhD candidates with the experience in conducting qualitative research, including focus groups and interviews.

The researchers carefully developed the focus group and had an interest and insight into the research topic. Author 3 facilitated the group discussion. Managing the focus group session included delivering a prepared introduction, welcoming the interviewees, overviewing the topic, answering questions, and beginning the discussion by asking an open-ended question. In the focus group interview, the interviewers were familiar with the participants. In healthcare studies, familiarity between researchers and participants can be helpful because the ‘warm up’ time is short and thus the interview can be started rapidly. The interviews continued until data saturation, i.e. “sampling to redundancy” [24]. In our study, data saturation occurred when no new concepts, categories, or dimensions emerged [24]. The interviewers attempted to minimize bias as much as possible while interviewing and analyzing data using several techniques, such as bracketing, external checking, and keeping detailed records.

### Data analysis

Data analysis was carried out simultaneously with data collection [22] using the conventional qualitative content analysis method developed by Elo and Kyngas [27]. This method consisted of three phases of preparation, organization, and reporting. In the preparation phase, meaning units were extracted in the form of initial codes. In the organization phase, codes with similar meanings were labeled with one category; related categories were classified into a main category. The data was managed using the MAXQD software V. 2007.

### Trustworthiness

We applied four criteria proposed by Lincoln and Guba to evaluate trustworthiness, including credibility, dependability, conformability, and transferability [22]. Triangulation in the data collection and analysis, prolonged engagement (6 months), and maximum variance in sampling was performed to increase the data’s credibility. Moreover, member checking was considered by giving some of the developed codes to the participants to assess the degree of consensus on the codes among the researchers and the participants. Parts of the transcripts, along with the extracted codes and categories, were sent to two external examiners to assess the data analysis process and provide dependability. Transferability was achieved through providing rich descriptions [22, 24].

## Results

The participants included 11 women and 2 men. Demographic characteristics of the participants are summarized in Table 1. The content analysis led to the extraction of two main categories: “providing infrastructures” and “DNP as an opportunity to make positive outcomes.“ Table 2.

**Table 1:** Demographic characteristics of participants

Gender: Male( 2), Female (11)	
Age: Between 27 to 52	
Highest education level: PhD	
Rank:	
Faculty member, PhD (4)	
PhD candidate (9)	
Years of clinical experience: Mean =5	
Years of educational experience: Mean =12

**Table 2:** Main categories, categories and subcategories the establishing a DNP program from experts’ views

Main categories	***Categories***	***Subcategories***
***1. Providing Infrastructures***	1.1 Basic infrastructures	Need assessment in different levels Adequate resources Defining the role and scope of graduates Evaluation factors facilitating or inhibiting this transition using DNP executive countries’ experiences. Cultural adaptations
	1.2 Challenges of developing a DNP program without providing infrastructures	Graduates’ frustration after the unsuccessful DNP performance. Waste time and costs. Lack of guarantees for patient outcomes Inadequate support for promoting of profession Burden on the healthcare system Blind imitation of western countries
***2. DNP: As an Opportunity to Drive Positive Outcomes***	2.1 DNP to meet the patients’ newly emerging needs	Application of knowledge to practice Development of clinical nursing practice Technological advances Patients’ changing needs Healthcare systems’ complexity
	2.2 Improvement of patient outcomes	Evidence-based practice development Decrease in the theory – practice gap Advancement nurses’ roles

### 1- “Provide infrastructures”

Most of the participants considered the adequate provision of infrastructure resources essential before establishing DNP. This theme yielded the two following categories:

1–1. “Basic infrastructures”.

Insufficient basic infrastructures were considered among the significant concerns for transition to DNP and included the following subcategories: need assessment in different levels, adequate resources (costs, trained professors, physical environments in hospitals and colleges), role/position definition, salary, evaluation factors facilitating or inhibiting this transition using DNP executive countries’ experiences, and cultural adaptations.

In this regard, the participants stated:

*“I think infrastructure must first be provided. The roles of DNP nurses should be clearly defined; otherwise, it can lead to a big challenge” (# 4)*.

*“We require the need assessment program at colleges, hospitals, and the community levels” (# 11)*.

*“The DNP setup should be based on the needs and context of the Iranian community.” (# 10)*.

*“At present, Ph.D. graduates do not have a suitable passion in clinical settings. Definition of the new program is ambiguous….” I do not think the DNP launch will be successful. Even cultural adaptation is important”. (# 3)*

*“The fact that the DNP program exists only in developed countries is not a sufficient reason for the establishment of the DNP program in Iran. The proliferation of DNP degree programs in some developed countries has created a series of challenges and confusions …” (# 5)*.

1–2. “Challenges of developing a DNP program without providing infrastructures”.

From the participants’ views, graduates’ frustration after unsuccessful DNP performance, waste time and costs, a lack of guarantees for promoting patient outcomes, inadequate support for promoting the profession, the burden on the healthcare system, and blind imitation of western countries formed subcategories.

**“** *The DNP is not a certain solution for ensuring the quality of care, and the unsuccessful implementation will lead to the graduates’ frustration”. (# 9)*

*“The unplanned implementation of the DNP will lead to wasting costs and time” (# 2)*.

### 2. “DNP as an opportunity to drive positive outcomes”

The second theme yielded the two following categories:

2.1. “DNP to meet the patients’ newly emerging needs”.

Application of knowledge to practice, development of clinical nursing practice, technological advances, patients’ changing needs, and healthcare systems’ complexity are critical factors in improving specialized care. Weaknesses of Ph.D. programs in improving clinical competency in graduates were another critical concern among the participants.

*“Nurses need to achieve a more significant role in the clinical settings and policies. One of the weaknesses of the Ph.D. program is a theory-based curriculum. Graduates increase their distance from the clinical settings by upgrading their degree.” (# 13)*.

*“I believe nurses with practice doctorate can apply knowledge in a practice to solve the problems.” (#6)*.

2–2. “Improvement of patient outcomes”.

This category included three subcategories: evidence-based practice development, decrease in the theory–practice gap, and advancement of nurses’ roles. Evidence-based practice is the gold standard for safe care provision. This approach has a significant role in improving patient outcomes and reducing the theory-practice gap.

*“Evidence-based nursing can bridge the gap between research findings and clinical practice.” (# 12)*.

*“…No doubt, we need the DNP because one of the major challenges in nursing education is the theory-practice gap. Maybe, this strategy helps to improve quality of care” (# 1).*

## Discussion

This study explored experts’ views on the necessity of establishing a DNP program in Iran. Although DNP-prepared nurses may promote the profession at the leadership level and improve the quality of care and patient safety, they have an evident concern about their management and organization in clinical settings. Our findings revealed that insufficient infrastructures were essential obstacles to establishing a DNP program in Iran. Insufficient infrastructures were mainly related to need assessment, resources (costs, trained professors, and physical environments in hospitals and colleges), role/position definition, salary, cultural adaptation. The Iranian Medical Education Context is physician-centered, and nurses do not have enough authority to drive positive changes in healthcare settings. This finding is similar to that of the study conducted by Vahedian-Azimi et al. [10]. The results showed that poor nursing practice environment and insufficient resources [12], inadequate support for involvement in evidence-based practice, organizational barriers, poor decision-making, a lack of motivation for applying evidence in practice, and preference to follow the doctor’s orders were the most critical challenges in this field [28–30].

Ambiguity in DNP graduates’ role was another significant concern. The findings are consistent with those of Udlis and Mancuso [31]. Multiple areas of ambiguity concerning DNP-prepared nurses’ role existed in research, academia and academic leadership, and scholarship.

Transition to a practice doctorate level will be expensive regarding resources and other factors. McCauley et al. [32] stated that financial challenges were the greatest obstacle to DNP acceptance of a doctoral program. Indeed, shifting to a new paradigm to solve DNP problems is not achievable only by DNP without providing foundations. A DNP program is only one piece of the puzzle in solving these issues. Stoeckel and Kruschke [33] assessed DNP-prepared NPs’ perceptions of the DNP degree, and reported that although DNP programs improved health population, evidence-based practice, research, and healthcare policies, it did not result in any significant progress in clinical skills. Participants mentioned laws and regulations as barriers to the full practice of DNP-prepared nurses.

In contrast, recommending the DNP degree without focusing on research is not applicable, which is a challenge for DNP executive countries. It is essential to equip DNP students with clinical research skills, quality advancement competencies, and opportunities to apply knowledge in the practice of desirable healthcare delivery [19]. Therefore, DNP programs and curriculums must be refined and reformed [19, 34]. Ketefia and Redman [35] in their study reported, although DNP has been conceived as a practice-focused degree, graduates are mainly taking faculty positions, for which they have not been trained, and a smaller number is seeking leadership roles. The exponential growth in the number of DNP programs in the United States has raised significant concerns about the discipline’s continuing ability to build a body of knowledge at a reasonable rate [5].

On the other hand, failure to apply the knowledge generated by Ph.D. graduates and improve care quality has been the motive to fill this gap. Brown and Crabtree [36] noted that doctoral education was undergoing a paradigm shift that needed more rapid improvement of practice expertise. They proposed creating DNP to achieve improved clinical practice and translating evidence into practice. Although our findings showed that infrastructure provision was an essential component in establishing DNP, “DNP as an opportunity to drive positive outcomes” was extracted as the second theme. Some of the participants supported the DNP degree as an appropriate degree to provide advanced clinical practice and leadership and redesign the healthcare system. The participants believed that specialized care should be improved due to technological advances, healthcare systems’ complexity, and changing patient needs. In line with our findings, Feizolahzadeh et al. [16] reported that Ph.D. graduates in Iran had excellent qualifications in education and research, but were unable to meet the needs of clinical settings, focusing on specialized clinical roles. Therefore, employing Ph.D. graduates in clinical settings will waste their time, energy, and expertise. They suggested designing and implementing DNP programs for advanced evidence-based practice.

Murphy et al. [1] believed that the collaboration between DNP and PhD-prepared nurses to advance the science and translation of research into practice provided educational opportunities, facilitated healthcare delivery, and resulted in positive outcomes. As clinical leaders, DNP-prepared nurses can play an essential role in changing health systems and improving specialized care, evidence-based practice, and patient outcomes [37].

### Limitation

Due to the qualitative design of the study with purposive sampling, generalization of research findings to the larger population is limited.

## Conclusions

It is concluded that it is not necessary to establish a DNP program for Iran’s nursing education system. Supplying infrastructures is a crucial component to establishing a DNP program in Iran. Inadequate resources, as well as cultural, social, economic, and political differences in developing countries, especially Iran, compared to developed countries, are significant limitations in implementing DNP. Therefore, a new program should be developed more carefully based on the health system’s needs, the society’s requirements, and doctoral candidates’ expectations. Before shifting to a new paradigm, its advantages and disadvantages should be carefully evaluated and criticized in each society’s cultural context. Although DNP, as an opportunity to drive positive changes, is recommended in nursing education, in the current situation, using alternative solutions may yield better outcomes than establishing a DNP program. In this regard, revising the curriculum, developing MSN levels, and modifying and refining the Ph.D. program, such as creating fellowship programs with a focus on clinical practice, may improve patient outcomes and decrease the theory-practice gap.

### Recommendation

Currently, the COVID-19 pandemic has highlighted the valuable role of physicians and healthcare providers, especially front-line nurses, in saving patients’ lives in Iran and around the world. In developed countries, DNP nurses may play an essential role in resolving this global crisis through evidence-based practice, critical thinking, resource management, health economics, health technology, and leadership at the national and international levels. Therefore, due to insufficient evidence on this issue, it is recommended to address DNP nurses’ specialized role with respect to the novel coronavirus in future studies.

## Supplementary information

**Additional file 1.**

## Data Availability

The raw data of the current study are not publicly available due this study is a qualitative research but are available from the corresponding author on reasonable request.

## Abbreviations

DNP: Doctor of Nursing Practice
Ph.D.: Doctor of Philosophy
MSN: Master of Science degree in nursing
AACN: Association of Colleges of Nursing
COVID-19: Corona Virus Disease-2019
