# Correction to: Exploring the necessity of establishing a doctor of nursing practice program from experts’ views: a qualitative study

**DOI:** 10.1186/s12909-021-02828-z

**Published:** 2021-07-24

**Authors:** Mozhgan Rivaz, Paymaneh Shokrollahi, Elahe Setoodegan, Farkhondeh Sharif

**Affiliations:** 1 Community Based Psychiatric Care Research Center, Department of Nursing, School of Nursing and Midwifery, Shiraz University of Medical Sciences, Shiraz, Iran; 2 School of Medical Science, Islamic Azad University- Firoozabad, Firoozabad, Iran; 3 Student Research Community, Iranian Social Security Organization, Shiraz University of Medical Sciences, Clinical Supervisor of Beheshti Hospital, Shiraz, Iran; 4 Community Based Psychiatric Care Research Center, Shiraz University of Medical Sciences, Shiraz, Iran

**Correction to: BMC Med Educ 21, 328 (2021)**

**https://doi.org/10.1186/s12909-021-02758-w**

Following publication of the original article [1], the authors identified an error in the #2 affiliation.

The correct affiliation has been updated above.

Also, they would like to correct the **Methods** section.

The updated section is given below and the changes have been highlighted in **bold typeface**.

**Trustworthiness**

We applied four criteria proposed by Lincoln and Guba to evaluate trustworthiness, including credibility, dependability, conformability, and transferability [22]. **Triangulation in the data collection and analysis, prolonged engagement (6 months), and maximum variance in sampling was performed to increase the data’s credibility**. Moreover, member checking was considered by giving some of the developed codes to the participants to assess the degree of consensus on the codes among the researchers and the participants. Parts of the transcripts, along with the extracted codes and categories, were sent to two external examiners to assess the data analysis process and provide dependability. Transferability was achieved through providing rich descriptions [22, 24].

The original article [1] has been corrected.
