# Supplementary material for: Exploring the necessity of establishing a doctor of nursing practice program from experts’ views: a qualitative study
Source: BMC Med Educ. 2021 Jun 7;21:328. doi: 10.1186/s12909-021-02758-w (PMC8183583; doi:10.1186/s12909-021-02758-w)
Supplement: Supplementary file 1 — Additional file 1. [file 12909_2021_2758_MOESM1_ESM.docx]

**Interview guide questions**

1. What do you think about setting up the DNP program in the Iranian nursing education system?
2. Can you explain the weakness of the Ph.D. Program or postgraduate programs?
3. What are the major barriers of the DNP implantation in your opinion?
4. What would be the consequences of the failure of setting up the DNP program in your opinion?
5. In the current situation, what is an appropriate postgraduate program for developing clinical competencies?
6. What would be the advantages of establishing the DNP program?
7. Do you have any additional suggestions that might clarify your agreement or opposition toward the establishing of the DNP program in Iran?
